# Influence of continental history on the ecological specialization and macroevolutionary processes in the mammalian assemblage of South America: Differences between small and large mammals

**DOI:** 10.1186/1471-2148-8-97

**Published:** 2008-03-26

**Authors:** Ana Moreno Bofarull, Antón Arias Royo, Manuel Hernández Fernández, Edgardo Ortiz-Jaureguizar, Jorge Morales

**Affiliations:** 1 Dept. Biología, Facultad de Ciencias, Universidad Autónoma de Madrid. C/Darwin 2, 28049 Cantoblanco, Madrid, Spain; 2 Serv. Microbiología, Hospital Universitario Ramón y Cajal. Cr. Colmenar Viejo Km 9, 28034 Madrid, Spain; 3 Dept. Paleontología, Facultad de Ciencias Geológicas, Universidad Complutense de Madrid. C/José Antonio Novais 2, 28040 Madrid, Spain; 4 Unidad de Investigación de Paleontología, Instituto de Geología Económica, Consejo Superior de Investigaciones Científicas. C/José Antonio Novais 2, 28040 Madrid, Spain; 5 Laboratorio de Sistemática y Biología Evolutiva (LASBE), Facultad de Ciencias Naturales y Museo, Universidad de la Plata. Paseo del Bosque s/n, B1900FWA La Plata, Argentina; 6 Dept. Paleobiología, Museo Nacional de Ciencias Naturales, Consejo Superior de Investigaciones Científicas. C/José Gutiérrez Abascal 2, 28006 Madrid, Spain

## Abstract

**Background:**

This paper tests Vrba's resource-use hypothesis, which predicts that generalist species have lower specialization and extinction rates than specialists, using the 879 species of South American mammals. We tested several predictions about this hypothesis using the biomic specialization index (BSI) for each species, which is based on its geographical range within different climate-zones. The four predictions tested are: (1) there is a high frequency of species restricted to a single biome, which henceforth are referred to as stenobiomic species, (2) certain clades are more stenobiomic than others, (3) there is a higher proportion of biomic specialists in biomes that underwent through major expansion-contraction alternation due to the glacial-interglacial cycles, (4) certain combinations of inhabited biomes occur more frequently among species than do others.

**Results:**

Our results are consistent with these predictions. (1) We found that 42 % of the species inhabit only one biome. (2) There are more generalists among species of Carnivora than in clades of herbivores. However, Artiodactyla, shows a distribution along the specialization gradient different from the one expected. (3) Biomic specialists are predominant in tropical rainforest and desert biomes. Nevertheless, we found some differences between small and large mammals in relation to these results. Stenobiomic species of micromammalian clades are more abundant in most biomes than expected by chance, while in the case of macromammalian clades stenobiomic species are more frequent than expected in tropical rainforest, tropical deciduous woodland and desert biomes only. (4) The most frequent combinations of inhabited biomes among the South American mammals are those with few biomes, i.e., the ones that suffered a higher rate of vicariance due to climatic cycles.

**Conclusion:**

Our results agree with the resource-use hypothesis and, therefore, with a major role of the past climatic changes as drivers of mammalian evolution. Nevertheless, deviations from the expectations indicate the importance of differences in reproductive traits and paleobiogeographic history for the macroevolutionary processes involved. In the case of South American mammals, the Pliocene Great American Biotic Interchange strongly influences the ecological characteristics of this assemblage. Furthermore, the Andes have acted as a fertile ground for speciation in environments prone to vicariance. Finally, the micromammals appear as more prone to biomic specialization than larger species. These factors are responsible for some of the differences found between South America and Africa in the studied pattern. For example, the extensive South American mountain ranges favour a higher number of combinations of inhabited biomes in comparison with Africa.

## Background

There are important connections between the global patterns on species biodiversity and current environmental conditions, but they are also influenced by past continental geography and the macroevolutionary processes determining speciation and extinction. According to the habitat theory on macroevolution [1-3], the main promoters of speciation and extinction are physical environmental changes (tectonics and climatic changes), instead of biotic interactions. Vrba's resource-use hypothesis [4,5], which is included as a part of this theory, stresses the role of the degree of specialization in biome-specific resources on the differences in speciation and extinction rates among clades. Generalists are less susceptible to withdrawal of their resources, to strong directional selection and to vicariance as environments change. This causes lower speciation and extinction rates in generalist species, while they are higher in specialists, which are converse in all these respects. The term resource covers a wide range of physical and biotic factors including moisture, temperature, substrate, vegetation cover, food items, and any other environmental components than can be utilized by organism [5]. The resource-use hypothesis differs from others regarding how the character "specialist" or "generalist" in a species is related to its distribution on terrestrial biomes. A species will be considered stenobiomic or eurybiomic according to the number of biomes it is able to inhabit, which are characterized by gross vegetation physiognomy. Thus, a stenobiomic species is restricted to a particular biome, or narrow range of vegetation physiognomy, and its lineage is predicted to have a high speciation rate if it suffers vicariance due to an environmental change that fragments the distribution of that biome. On the contrary, a linage of eurybiomic species shows a lower speciation rate because it can use resources in more than one biome and, therefore, it is not severely affected by fragmentation of the biomes it inhabits. Under this hypothesis, the faster rate of speciation in stenobiomic species generates an average bias towards overrepresentation of biome specialists in clades and ecosystems over long time periods [5,6].

The resource-use hypothesis was originally conceived by Vrba in 1987 [5] after the study of the African fossil record on large mammalian clades. However, the information known on the fossil record of certain biological groups or geographical areas is far from complete. That is why Hernández Fernández and Vrba in 2005 [7] used the modern assemblage of African large mammals to test this hypothesis. Their analyses appeared to offer support for each of four subsidiary predictions of Vrba's resource-use hypothesis: (1) since clades of biomic specialist species generally have had a high incidence of vicariance, speciation and extinction, these species should be clearly more numerous than eurybiomic species; (2) certain clades should be more eurybiomic than others because the resources they need to survive may be found in environments which differ vastly in climate; (3) biomes that underwent a high degree of fragmentation during the recurrent environmental extremes of the climatic cycles should have a higher proportion of stenobiomic species than those that have not undergone extensive fragmentation; and finally (4) from the previous prediction, it should be expected that certain combinations of inhabited biomes occur more frequently among species than do others. These combinations must be those that include few biomes [7].

In this work we tested these predictions about the resource-use hypothesis using the biomic specialization index (BSI) for the complete assemblage of South American land mammal species. This measure of ecological specialization, based in the number of inhabited biomes by each species (Table 1), is used here because it is useful in intercontinental and intertaxa comparisons [7]. Finally, we compared our results with those presented by Hernández Fernández and Vrba [7] on the large mammals of Africa in order to find some clues into the macroevolutionary processes responsible for the differences found between these continents. These are the two largest tropical continental masses, and both share similar climatic zonations and have a large number of mammal species despite of their very different evolutionary history after the fragmentation of Gondwanaland. While Africa has maintained frequent terrestrial connections with Eurasia during the last 20 million years [8], South America was an isolated land mass which had no connections with other continents since the final opening of the Drake Passage ca. 30 Ma, which separated it from Western Antarctica, until the Late Pliocene, when the Panamanian land-bridge appeared [9]. Since that time, there was an unparalleled and continuous biotic interchange between South and North America, the so called Great American Biotic Interchange (GABI) [10-14]. Many mammal groups migrated from one land mass into the other, although the final outcome of this event appears to be favorable to the groups of North American origin. This exchange produced significant changes in the structure of the South American mammal communities [15,16]. The partial replacement of South American autochthonous species by North American immigrants has been attributed to three different reasons. First, during the GABI new ungulates and carnivores arrived to South America. The native ungulates (notoungulates, litopterns, pyrotheres, etc.) declined under pressure from novel, placental predators [10]. Meanwhile, the northern ungulates that moved to the new continent found new habitats with the resources they needed [12]. Second, the unbalanced species interchange between the Americas was caused by the disparity in richness of the original species pools due to the differences in size of both continents [17]. The equilibrium theory of island biogeography [18] predicts a migration wave favorable to the North American immigrants, which came from a larger continent with a larger species pool [17]. Third, the reason for the unbalance of the GABI has been also placed in the global climatic changes that produced variations in the environments from the isthmian Central America [1,19]. While during the warm and humid periods the tropics were dominated by rainforests, in colder and more arid phases savanna habitats extended broadly through tropical latitudes. Therefore, as Cenozoic climate was getting colder a savanna corridor became increasingly stable between North and South America while the extension of tropical forest shrunk. At the time of the GABI, when modern glacial cycles were established, only generalist species or species specialists of the savanna biome were favored when crossing the corridor between both continents [1]. This latter explanation of the unbalanced result of the GABI may be related to the outcome of some of the predictions of the resource-use hypothesis on the current mammal fauna of South America.

**Table 1:** Climatic typology of Walter [66] and it's correspondence with world vegetation types

	Climate zone	Zonobiome
I	Equatorial	Evergreen tropical rain forest
II	Tropical with summers rains	Tropical deciduous woodland
II/III	Transition tropical semiarid	Savanna
III	Subtropical arid	Subtropical desert
IV	Winter rain and summers drought	Sclerophyllous woodland-shrubland
V	Warm-temperate	Temperate evergreen forest
VI	Tropical temperate	Nemoral broadleaf-deciduous forest
VII	Arid-temperate	Steppe to cold desert
VIII	Cold temperate (boreal)	Boreal coniferous forest (Taiga)
IX	Artic	Tundra

An additional difference between South America and Africa it is based on the incidence that the late Quaternary extinction event had on their mammalian faunas. During the latest Pleistocene-earliest Holocene, land-mammal faunas all around the world changed as a consequence of the so called "megafaunal extinction", an extinction event that mainly affected large mammals. Nevertheless, in spite of its world-wide extension, this event affected on a larger extent the mammalian faunas of North America, South America, and Australia [20-23]. Several hypotheses have been proposed for explain this megafaunal extinction but none has been recognized yet as having larger support than the others [24-31]. According to Cione et al. [30], in South America 80 % of those mammal species weighting over 44 kg (e.g., the horses *Equus neogeus *and *Hippidion principale*, the mylodontid *Mylodon ibseni*, and the bear *Arctotherium bonariense*), and 100 % of those mammal species weigthing over 1000 kg (e.g., the toxodontid *Toxodon platensis*, the ground sloth *Megatherium americanum*, and the camel *Hemiauchenia paradoxa*) became extinct. Consequently, mega-mammals (species > 1000 kg) do not exist in the present-day South American land-mammal fauna, and only some individuals of the tapirid *Tapirus bairdii *weighting over 300 kg [32,33]. Conversely, five mega-mammal species and several large mammal ones occur in Africa today [32,34]. Most South American large mammals extinct during this event were grazers or mixed-feeders (e.g., equids, camelids, notoungulates, glyptodonts) and only a few were browsers (e.g., megatheriids). Most of them, specially the mega-mammals, had low abundance, and females probably attained sexual maturity late and had a low number of offspring, born after a very long gestation time, i.e. they were k-strategist [30]. Thus, according to the record of disappeared species, it seems that although this event was mainly modulated by species size the ecology of these species could have had a certain role in it. Thus, since in this work we are dealing mainly with the ecological specialization degree of the species, we will discuss whether the megafaunal extinction could have any significant influence in the patterns shown here.

## Results and discussion

### Distribution of the biomic specialization index (BSI) in South America

**Figure 1:**
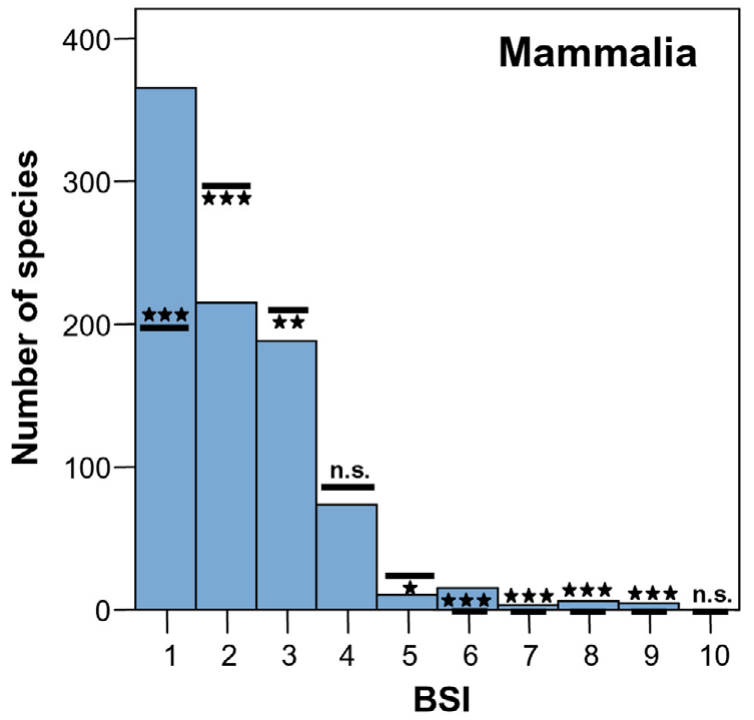
**Frequency distribution of biomic specialization index (BSI) for South American mammals**. The lines show the average number of species (± 2 S.E.) for each BSI calculated on 1000 Monte Carlo simulations (Table 2). ***, p < 0.001; **, 0.01 > p > 0.001; *, 0.05 > p > 0.01; n.s., not significant.

The general frequency distribution of BSI by South American mammals is strongly right-skewed (Figure 1). Mean BSI is 2.16. We found that 65.8 % of species inhabit only one or two biomes (BSI = 1, 41.7 % and BSI = 2, 24.1 %). At the other extreme, only a few species inhabits five or more biomes (4.32 %) and none occupied all biomes. To occupy all extreme biomes require a very high degree of versatility, which probably precludes a species from occupying all biomes, as already reported Hernández Fernández & Vrba [7]. Thus, most South American mammal species live in a narrow range of ecological conditions (represented by biomes). Our assemblage shows a significantly higher proportion of biomic specialist species (BSI = 1) than expected by a random process as modeled by Monte Carlo analysis (Table 2). The proportions of species with BSI = 2–4 are significantly (or nearly significantly for BSI = 4) lower than estimated by the Monte Carlo model. Although significantly lower, the proportion of species with BSI = 5 is not very different from than expected from the modeled random distribution of species in biomes. Finally, the proportions of species with BSI = 6–9 (there are no South American mammals with BSI = 10) are significantly higher than expected from the null hypothesis (Table 2). These results are broadly consistent with the resource-use hypothesis: a higher proportion of biomic specialist species than generalists. We also found that the proportions of extreme eurybiomic species are higher than expected by a random process. This coincides with the results of Hernández Fernández and Vrba [7] on the African large mammals. These authors argued that extreme eurybiomic species, thanks to their versatility, can survive in the biomes at both climatic extremes of the Milankovitch cycles, which are the cyclical variations in the Earth's eccentricity, axial tilt and precession, and primary cause of the episodic nature of the Earth's climate. These eurybiomic species, therefore, may have proportionally lower extinction rates than semi-eurybiomic species. Thus, the extreme eurybiomic species may have experienced a net increase in species over time as suggested by Hernández Fernández and Vrba [7].

**Table 2:** Proportion of South American mammals species in each BSI and comparison with the Monte Carlo simulations

BSI	%	Monte Carlo Analysis			
		
		Mean %	Std.dev	Range	*p*
1	41.54	23.10	1.21	19.18–27.24	< 0.001
2	24.40	34.51	1.64	29.17–39.05	*< 0.001*
3	21.34	24.76	1.29	20.54–28.94	*0.004*
4	8.29	9.50	5.84	6.92–12.26	*0.083*
5	1.14	2.02	23.10	0.57–3.52	*0.031*
6	1.70	0.25	34.51	0.00–0.91	< 0.001
7	0.45	0.01	24.76	0.00–0.23	< 0.001
8	0.68	0.00	9.50	0.00–0.11	< 0.001
9	0.45	0.00	2.02	0.00–0.00	< 0.001
10	0.00	0.00	0.25	0.00–0.00	1.000

N = 1000 simulations. %; proportion of the total number of species (879), *p*; probability of the proportion of species being greater than or equal to (plain) or lower than or equal to (italics) the observed proportion in the South American mammal fauna.

### Distribution of BSI in mammalian clades

**Figure 2:**
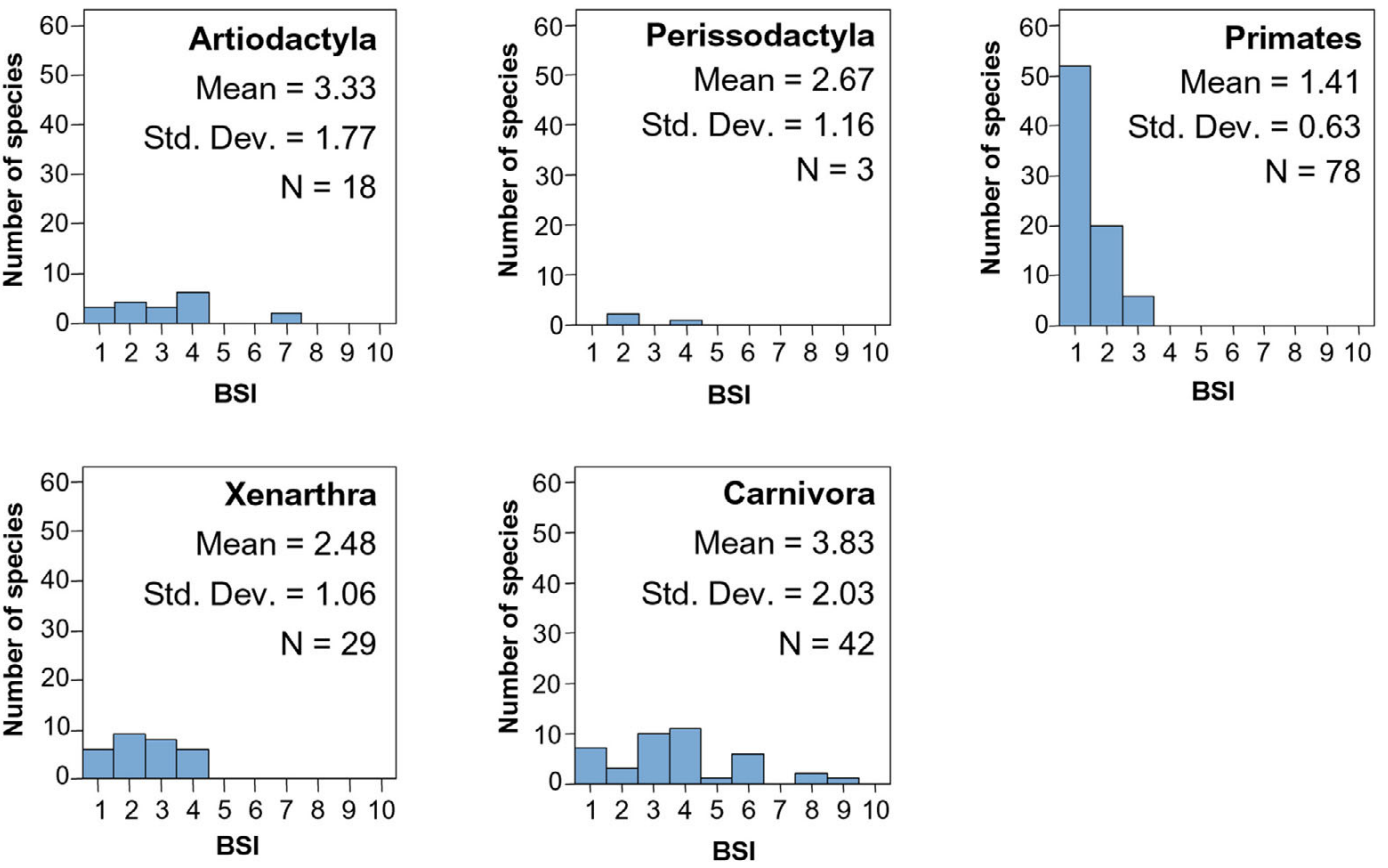
BSI histograms for macrommamalian orders.

**Figure 3:**
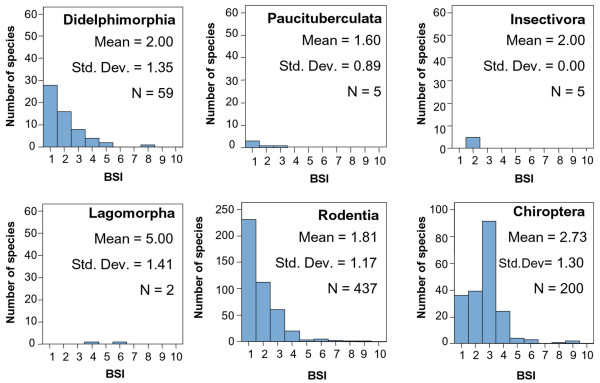
**BSI histograms for micromammalian orders**. Note the change in the vertical scale for Rodentia and Chiroptera. Microbiotheria is not included because it has only one species.

Table 3 shows the mean BSI value for each South American mammal group. Figures 2 and 3 compare the BSI histograms among mammalian orders.

**Table 3:** Mean BSI value and evolutionary group of each South American mammalian order.

ORDEN	n^o^ sp	Mean BSI	Evolutionary group
**Micromammalian**	709	2.45	
DIDELPHIMORPHIA	59	2.00	1
PAUCITUBERCULATA	5	1.60	1
MICROBIOTHERIA	1	2.00	1
RODENTIA	437	1.79	
Hystricognathi	157	1.71	2
Sciurognathi	280	1.88	3
INSECTIVORA	5	2.00	3
CHIROPTERA	200	2.74	
Emballonuroidea	17	2.47	2
Noctilionoidea	125	2.54	1
Vespertilionoidea	58	3.22	3
LAGOMORPHA	2	5.00	3
			
**Macromammalian**	170	2.74	
XENARTHRA	29	2.48	1
PRIMATES	78	1.41	2
CARNIVORA	42	3.83	3
PERISSODCTYLA	3	2.67	3
ARTIODACTYLA	18	3.33	3
			
Total	879	2.60	

No sp, species number. BSI, biomic specialization index. Evolutionary group: 1, South American autochthonous; 2, Eocene/Oligocene African immigrants; 3, Pliocene North American immigrants (the information of the evolutionary groups was obtained from [11,12,14,44,56,72,73]).

Carnivora is more eurybiomic and generalist than other clades (Figure 2), as suggested by the resource-use hypothesis [5]. Their feeding behavior is very characteristic; they can find their main food resource (meat) in environments which differ vastly in climate because generally they do not need a particular prey species [35].

Xenarthra is one of the most ancient orders in South America, and its histogram shows a distribution within one to four biomes (Figure 2). This is probably due to their differences in feeding. There are insectivore and omnivorous species, which show usually a more eurybiomic behavior, while herbivore species are more stenobiomic. Additionally, they show different locomotion adaptations. While Dasypodidae species are terrestrial and usually fossorial, Myrmecophagidae are terrestrial or scansorial, and Megalonychidae and Bradypodidae are arboreal. Obviously scansorial and arboreal species are linked to forest environments while terrestrial species do not have this limitation.

Distribution of BSI in Primates is as expected under the resource-use hypothesis, having a marked dominance of specialist species (Figure 2). It is an order whose species, which are either herbivores or insectivores-frugivores, are usually restricted to a particular biome. Additionally, they are also strongly related to forested landscapes.

Ungulates are herbivore species and they tend to be specialists restricted to a particular vegetation physiognomy. Therefore, according to the resource-use hypothesis, they should be stenobiomic [7]. Today there are only two South American orders of ungulates, Artiodactyla and Perissodactyla, both immigrants from North America during the Great American Biotic Interchange (GABI). The species of both orders are displaced from the specialist extreme of the stenobiomic-eurybiomic gradient (Figure 2). This might be explained because of their very diverse feeding, including fruit, leaves and grasses [32]. Vrba [5] predicted that organisms that both graze and browse, or are omnivores, are likely to be eurybiomic.

The generalist ecological behavior of these ungulates might be due to the biogeographic and evolutionary history of the South American mammalian assemblage and could be related with the differences in evolutionary success of the native southern ungulates and the northern immigrant ungulates. Four hypothesis proposed in the literature might be possible explanations for this generalist pattern in ungulates:

(1) There was a savanna corridor between both continents during the GABI. Only generalist species or species specialists of the savanna biome could cross the corridor between North and South America [1]. Many savanna specialist species crossed from North to South America because this biome was abundant in the Northern hemisphere. Also North American generalist species crossed it. On the other hand, the appearance of steppe and grassland habitats in this period reduced the number of native ungulate species [11] and few of them crossed into North America.

(2) Some studies attribute the loss of large-sized mammal lineages, like native South American ungulates, to the decrease of open vegetation area from the late Pleistocene last glacial maximum to the Holocene climatic optimum [31,36,37].

(3) Under the *Blitzkrieg *hypothesis [38], the human colonization in the late Pleistocene was especially lethal for large species. Nevertheless, human predation of large South American mammals had a marginal role, affecting only to small isolated populations [31]. Brook and Bowman [39] support this idea of the human predator role worldwide, although the operational details remain uncertain.

(4) Finally, other hypotheses [30] explain that the extinction of large-sized mammals was due to the combined action of the climatic changes that reduced the areas covered by open vegetation during the latest Pleistocene-earliest Holocene, and the pressure exercised by the human hunters, who entered to the continent during the present interglacial.

Didelphimorphia has a lot of species and there is a predominance of specialists (Figure 3), despite their insectivorous diet. This might be due to their small body size and the characteristics associated to smaller species. Energetic and physiological constraints create a high degree of specialization in small species [40,41]. Additionally, small-bodied species disperse more slowly and with lower rate of successful establishment in a new area [42], and therefore they may come to occupy a smaller proportion of their inhabitable biomes. Finally, generation interval is correlated positively with body size [43], which could allow micromammal species to reach greater degrees of specialization in less time than macromammals.

**Figure 4:**
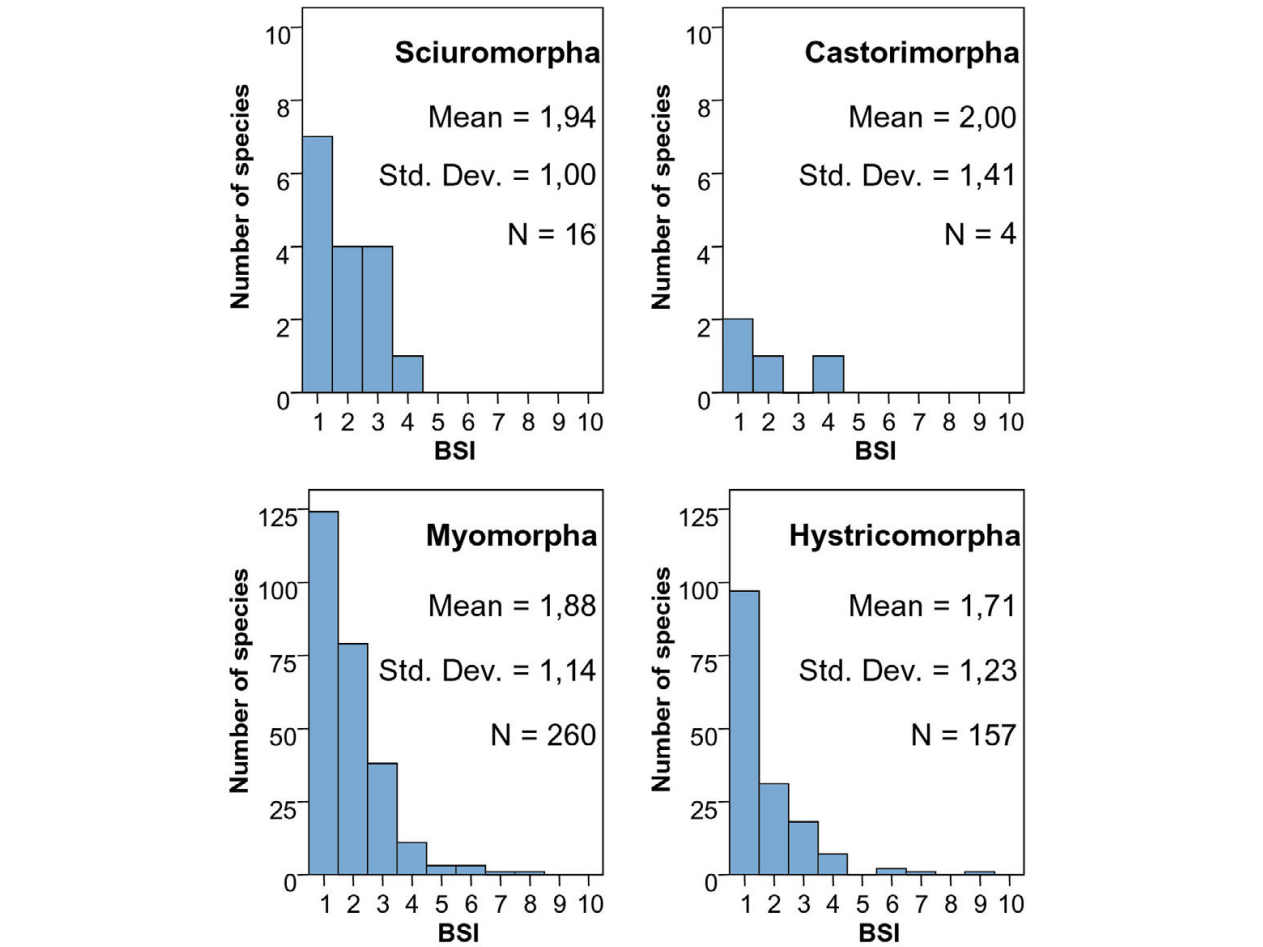
BSI histograms of Rodentia suborders [74].

The Rodentia frequency distribution of BSI is strongly right-skewed (Figure 3) due to the high number of species that inhabit in only one biome, which is probably related to their predominantly herbivore feeding. In this case, the difference in their evolutionary origin does not appear to influence the general trend of the order. The BSI proportions of each suborder of Rodentia (Figure 4) are very similar, although the American Hystricomorpha (caviomorphs) derived from Eocene/Oligocene African or Asian immigrants while the other suborders came from North America in the late Neogene. This is to say, they have a very similar distribution on the eurybiomic-stenobiomic gradient but the origins of their lineages are separated millions of years. However, focusing on the two main suborders, we should stress the fact that, although caviomorph and myomorph rodents are on average similarly specialized, there are relatively large differences in the number of species in these two groups. Hystricomorpha is an older inhabitant of the continent but now its species number is lower than in Myomorpha. This might be due to the size and reproductive behavior of species in these suborders. While Hystricomorpha includes the largest rodents in the world [11] and their growth rate is more similar to macromammals, Myomorpha has the typical growth rate of rodents. The higher reproductive potential of Myomorpha increases their evolutionary capacity. In this way, there are many South American species in the Myomorpha suborder in spite of their relatively recent colonization of the continent.

**Figure 5:**
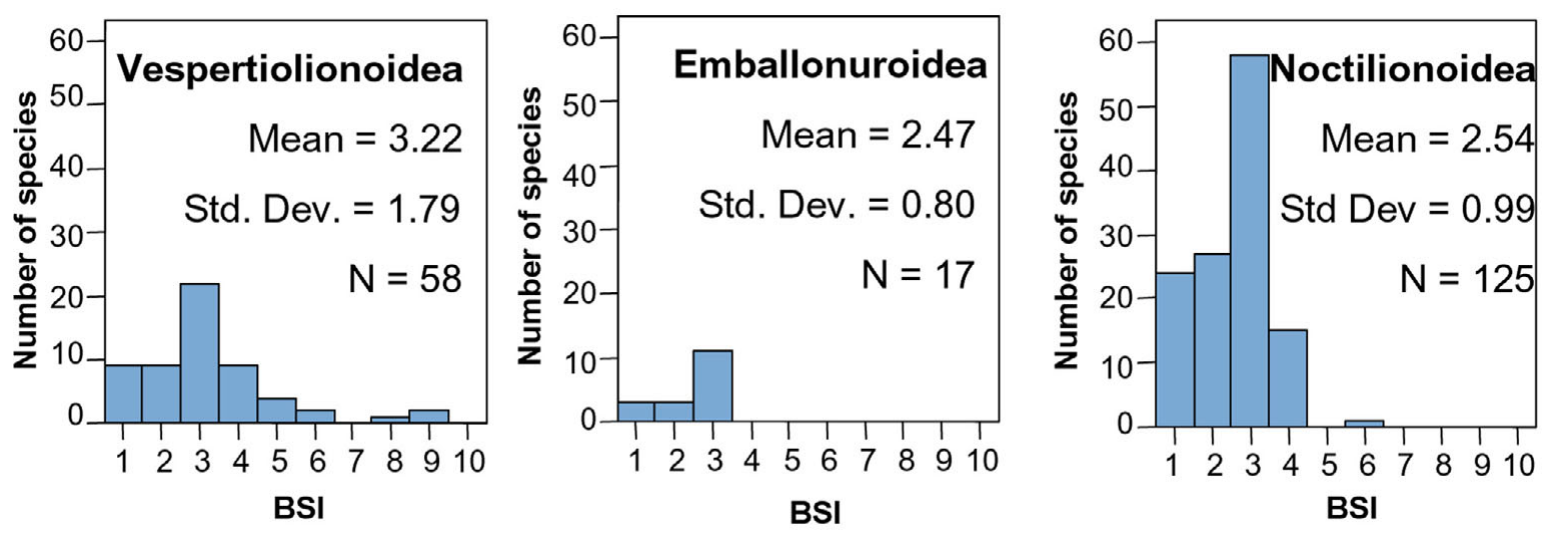
BSI Histograms of Chiroptera superfamilies.

Chiropterans are moderately generalists (Figure 3). Most of the species have a BSI = 3. Probably, this is due to their flight capacity, which allows them almost free movement between biomes. There are three superfamilian groups of this order in South America (Figure 5). Noctilionoidea and Emballonuroidea are eurybiomic groups that inhabit the Neotropical region although they also have some specialist species. Recent studies [44] suggest that Noctilionoidea, confined to the Neotropics and the most numerous group of bats in the continent, is autochthonous of South America. On the other hand, the origin of Emballonuroidea superfamily is in Africa, coinciding with the arrival of other taxa during the Eocene/Oligocene, and its distribution is exclusively tropical on opposite sides of the Atlantic. It is the bat group with less species, which could be due to its more recent arriving to the continent and interspecific competition with Noctilionoidea species. Vespertilionoidea is the chiropteran superfamily with the largest gaps in its known fossil record [44]. This superfamily is Laurasian in origin and, thus, it is included in the third evolutionary phase of the South American Mammals faunas, the groups immigrated from North America during the Pliocene (see Table 3). Vespertilionoidea shows the highest mean BSI of all the bat groups, which is probably related to their North American origin and recent colonization of South America.

**Figure 6:**
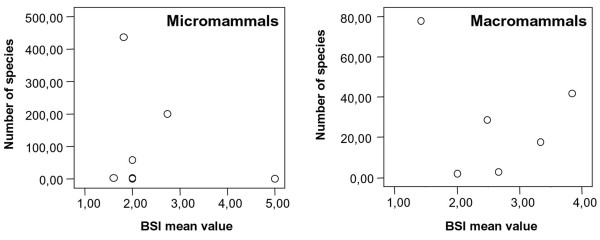
Relationship between BSI mean value and number of species in each clade of micro- and macromammals.

The first prediction of the resource-use hypothesis said that generalist species have lower speciation and extinction rates. Thus these species should be clearly less numerous than stenobiomic species. However there is no correlation between both variables (Figure 6). To be specialist or generalist species is not the only factor that determines the number of species in each clade. The emergence of the Panamanian land bridge and the climatic changes about 3 million years ago controlled what species crossed between both continents and the survival and adaptation of the native South American species. Furthermore the Andes ranges involve a series of altitudinal surfaces with strong tendency to vicariance due to climatic changes. Two other factors based in these arguments are very important to analyze our results. First, the evolutionary origin of the taxon must be taken into account. This factor is composed for many other subsidiary factors like, the unequal biotic interchange between continents and their consequences in immigrants and endemic species, etc [1]. Second, macromammals (MAC) and micromammals (MIC) have very different behavior in relation to biomic specialization. Tectonic and paleoenvironmental changes favor the MIC speciation due to their higher capabilities for fast adaptive radiation [1] and usually smaller ranges [45]. This argument obtains support when comparing the number of MAC and MIC species in Africa and South America in relation to the continental size. South America is smaller than Africa but there is almost no difference between the numbers of MIC in both continents, 773 species in Africa and 709 in South America. Thus, it seems that in spite of the smaller size of South America their topographic characteristics have favored a fast radiation in MIC species. Meanwhile the lower diversification rates in MAC orders are due to these tectonic and paleoenvironmental changes and the important contribution of the recent North American immigrants. The gradual pattern of extinction of large mammals species during the Pliocene and the late Quaternary megafaunal extinction event were strong in South America and almost all continents while it was restricted to some large mammals in Africa [46], allowing an increase in the richness of the rest of the large mammals [47]. Therefore, native large mammals are much more important herbivores in Africa than in South America. Finally, the post-Pleistocene differences between the abundances of large herbivore mammals on the two continents may be related to different frequencies of nutritionally sufficient habitats [48]. All this might explain the higher number of African MAC (250) and their differences with South America (170).

### Proportion of biomic specialists in each biome

The third prediction associated to the resource-use hypothesis states that biomes that underwent major cycles of expansion-retraction and a high degree of fragmentation during the recurrent environmental extremes of the astronomical cycles (Milankovitch cycles) should have a higher proportion of stenobiomic species (BSI = 1) than those that did not undergo extensive fragmentation. At the global scale, these biomes are located in extreme climatic conditions [7,49]: tropical rainforest (biome I), subtropical desert (biome III), steppe (biome VII) and tundra (biome IX). Nevertheless, at the continental scale the biomes that suffered such fragmentation may be different in each continent, depending on the presence/absence of these biomes, the biomes present at the geographical extremes of that continent, the internal heterogeneity of climatic dominions, or the biogeographic structure of the continent.

Table 4 shows the results for all South American mammals, which support this idea. We can see how the proportion of stenobiomic species (BSI = 1) is significantly higher than expected by chance in extreme biomes; tropical rainforest, subtropical desert and steppe. However, tropical deciduous woodland (Biome II, Table 1) and boreal coniferous forest (Biome VIII), which are not climatically extreme biomes, show also a significantly higher proportion of stenobiomic species than expected by chance. South America suffered about 2.5 Ma a cooling trend with glacier formation and a cycle of expansion and reduction of grasslands and forests [1], species specialists of these biomes should have high extinction and speciation rates. Tropical deciduous woodland is mainly located around the equator and along the Andes, sites that suffered strongly this process. Additionally, tectonic changes during the late Neogene caused the elevation of the Andes from 2000 to 4000 meters, which allowed the formation of different vegetation belts more o less continuous, analogous to the boreal coniferous forest (*Podocarpus *forest, VIII) or to the steppe (the montane grasslands so-called Puna, VII). Later on, during successive glacial periods, this Andean vegetation belts underwent extensive vicariance. Thus, the speciation rates increased in species that lived in these biomes [1].

**Table 4:** Stenobiomic number species (BSI = 1) in South American mammals

	Mammals						
	
Biome	South America			Monte Carlo analysis			
		
	sp.	sp. (BSI = 1)	%	Mean %	Std.dev.	Range	*p*
I	506	149	29,4	13,65	1,34	9,68–18,18	< 0,001
II	504	104	20,6	13,55	1,31	9,52–17,46	< 0,001
II-III	282	18	6,4	8,46	1,51	4,60–14,18	*0,051*
III	64	16	25,0	6,38	3,01	0,00–20,31	< 0,001
IV	39	3	7,7	6,16	4,01	0,00–23,08	0,279
V	227	24	10,6	7,86	1,69	3,08–14,10	0,146
VI	65	7	10,8	6,18	2,94	0,00–18,46	0,096
VII	111	22	19,8	6,66	2,30	0,00–14,41	< 0,001
VIII	105	24	22,9	6,57	2,39	0,95–14,29	< 0,001

sp., number of species; % proportion of species with BSI = 1 in relation to total number of species; *p*, probability in each biome of the proportion of species with BSI = 1 being greater than or equal to (plain) or lower than or equal to (italics) the observed proportion in the South American mammal fauna.

In order to compare our results with the previous ones for the African large mammals [7], we separated ours in macromammals (MAC) and micromammals (MIC) (Table 5). The proportion of macromammalian stenobiomic species in South America is significantly higher than expected by chance in biomes I, II, and III, which is comparable to the results for the African faunas [7]. Our results support the resource-use hypothesis because these biomes underwent extensive fragmentation during the cycles of climatic changes along the Cenozoic as stated above. The rest of the biomes show a number of stenobiomic large mammals that is not significantly different of the proportion that may be obtained by chance. This might indicate that those biomes did not undergo fragmentation extensive enough for vicariance and speciation events of large mammals. It could also be argued that the important late Quaternary event of megafaunal extinction might have affected this pattern. Nevertheless, nearly all the South American extinct large and mega-mammals were adapted to open environments, and many of them were even adapted to arid ones (see [30], and references therein). Therefore, they might have been included as representatives of the arid biomes in South America, which probably would give additional support to our conclusions. Anyhow, since large organisms are constrained to have relatively low population densities, in order to maintain a minimum viable global population large species require large geographic ranges [41] frequently across several biomes, and thus the proportion of strict stenobiomic species within the subset of large and very species is usually very small [34]. Therefore it would be difficult that these few biome specialist species could have statistical influence on our conclusions about the stenobiomic macromammals.

**Table 5:** Stenobiomic number species (BSI = 1) in South American macromammals and micromammals

	Macromammals						
	
Biome	South America			Monte Carlo analysis			
		
	sp.	sp. (BSI = 1)	%	Mean %	Std.dev.	Range	*p*
I	123	50	40.65	12.05	2.58	4.88–20.33	< 0.001
II	96	12	12.50	7.71	2.48	1.04–14.58	0.028
II/III	60	2	3.33	5.37	2.73	0.00–16.67	*0.358*
III	15	2	13.33	3.69	4.96	0.00–26.67	0.019
IV	12	0	0.00	3.92	5.29	0.00–33.33	*0.602*
V	52	1	1.92	4.88	2.84	0.00–15.38	*0.260*
VI	18	1	5.56	4.02	4.56	0.00–22.22	0.836
VII	22	1	4.55	3.85	4.16	0.00–22.73	0.795
VIII	15	0	0.00	3.86	4.77	0.00–26.67	*0.541*
							
	Micromammals						
	
Biome	South America			Monte Carlo analysis			
		
	sp.	sp. (BSI = 1)	%	Mean %	Std.dev.	Range	*p*

I	386	98	25.39	13.99	1.57	8.88–19.84	< 0.001
II	409	92	22.49	15.22	1.50	10.51–19.80	< 0.001
II/III	222	16	7.21	9.37	1.82	4.50–15.32	*0.150*
III	49	14	28.57	7.01	3.71	0.00–20.41	< 0.001
IV	27	3	11.11	6.62	4.67	0.00–25.93	0.096
V	175	23	13.14	8.43	2.02	3.43–16.00	0.006
VI	47	6	12.77	6.81	3.65	0.00–21.28	0.038
VII	89	21	23.60	7.32	2.84	1.12–21.35	< 0.001
VIII	90	24	26.67	7.38	2.68	0.00–16.67	< 0.001

sp., number of species; % proportion of species with BSI = 1 in relation to total number of species; *p*, probability in each biome of the proportion of species with BSI = 1 being greater than or equal to (plain) or lower than or equal to (italics) the observed proportion in the South American mammal fauna.

Nevertheless, in the case of small mammals all the biomes present in South America, except II/III and IV, showed significantly more stenobiomic species than expected after the Monte Carlo modeling. This included not only the hypothetical extreme biomes but also climatically transitional biomes like II, V, VI and VIII. Differences in the physiology, adaptations and ecology of small mammals may be responsible for the differences found between small and large mammals. For example, the proportion of specialist species for MIC in the deserts (III) is 28.57 %, which is double than for MAC (13.33 %). Desert biome requires a high degree of specialization in morphology, physiology and behavior, which smaller species probably can get faster than large mammals, due to their lower generation intervals [43]. Also, global climatic changes during the Plio-Pleistocene caused a higher degree of vicariance in the Andes region favoring the radiation of MIC orders, while MAC species had lower time to adapt and specialize themselves in these areas. In this sense, the number of stenobiomic species in the *Podocarpus *forest (Biome VIII) is very high in MIC (26.67 %), while there are no MAC species exclusive of this biome. Along the Andes MIC moved faster to the south of the continent than MAC and, according to Ortiz-Jaureguizar and Cladera [50], the southern steppes (VII) underwent several pulses of expansion and retraction, due to the glacier formation, just like the nemoral broadleaf-deciduous forest (VI) in relation to their position in the continent. Hence, our MIC results show a high degree of specialist species in theses biomes. The proportion of stenobiomic MIC species in biome V is also higher than expected by chance, which may indicate a trend to contraction-expansion and associated fragmentation for this biome. This result could be associated to the fact that the South American climatic dominions of this biome are mainly surrounded by biomes with a drought period, which might influence in the capabilities of the species inhabiting this biome to occupy other neighboring biomes.

### Climatic combinations

**Figure 7:**
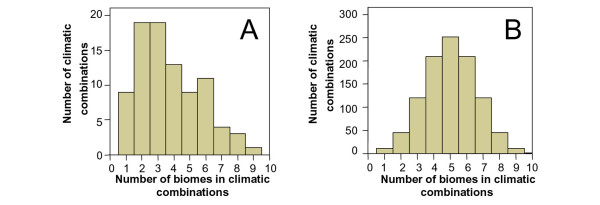
**Frequencies of climatic combinations, in terms of numbers of biomes inhabited by South American mammals species**. A, observed; B, potential.

We can see a substantial difference between the number and distribution of potential total climatic combinations (PTCC) and the actual climatic combinations present in the assemblage of all South American mammals (Figure 7). We found 88 climatic combinations of the 1023 potential climatic combinations (Table 6). Our results indicate that there are significantly more combinations of few biomes than expected in a random selection of combinations from the PTCC (χ^2 ^= 860.4, d_0_F = 9, p < 0.001).

**Table 6:** Climatic combinations in South America terrestrial mammals today

Climatic combinations	N^o^ biomes	Sp.
I	1	147
I-II	2	114
I-II-II/III	3	118
I-II-II/III-III	4	1
I-II-II/III-III-IV	5	1
I-II-II/III-III-IV-V	6	2
I-II-II/III-III-IV-V-VI-VII	8	2
I-II-II/III-III-IV-V-VI-VII-VIII	9	4
I-II-II/III-III-V	5	2
I-II-II/III-III-V-VI-VII	7	1
I-II-II/III-III-V-VII-VIII	7	1
I-II-II/III-III-V-VIII	6	1
I-II-II/III-III-VII	5	1
I-II-II/III-IV-V-VI-VII-VIII	8	1
I-II-II/III-IV-V-VII	6	1
I-II-II/III-V	4	54
I-II-II/III-V-VI-VII	6	1
I-II-II/III-V-VI-VII-VIII	7	1
I-II-II/III-V-VII-VIII	6	2
I-II-II/III-V-VIII	5	1
I-II-III-V-VII-VIII	6	1
I-II-V	3	26
I-II-V-VIII	4	2
I-II/III	2	1
I-II/III-V	3	4
I-V	2	11
I-V-VIII	3	2
II	1	104
II-II/III	2	23
II-II/III-III	3	2
II-II/III-III-IV-V	5	1
II-II/III-III-IV-V-VI-VII-VIII	8	3
II-II/III-III-IV-V-VII	6	1
II-II/III-III-IV-VI-VII	6	2
II-II/III-III-VII-VIII	5	1
II-II/III-IV-V-VII-VIII	6	1
II-II/III-V	3	4
II-II/III-V-VI-VII-VIII	6	1
II-II/III-V-VII-VIII	5	1
II-II/III-VII	3	1
II-II/III-VIII	3	1
II-III	2	1
II-V	2	12
II-V-VII-VIII	4	1
II-V-VIII	3	3
II-VII	2	1
II-VIII	2	1
II/III	1	18
II/III-III	2	1
II/III-III-IV	3	1
II/III-III-IV-V-VI-VII	6	1
II/III-III-IV-V-VI-VII-VIII	7	1
II/III-III-IV-VII	4	1
II/III-III-V	3	1
II/III-III-V-VI-VII-VIII	6	1
II/III-III-VI-VII	4	4
II/III-III-VII-VIII	4	1
II/III-IV-V-VI-VIII	5	1
II/III-V	2	4
II/III-V-VII-VIII	4	1
II/III-VI-VII	3	2
II/III-VII	2	2
III	1	16
III-IV	2	1
III-IV-V-VI	4	2
III-IV-VII-VIII	4	1
III-V-VI-VII	4	1
III-VI	2	1
III-VII-VIII	3	2
IV	1	3
IV-V-VI	3	5
IV-V-VI-VII	4	1
IV-V-VI-VII-VIII	5	1
IV-VI-VIII	3	2
V	1	24
V-VI	2	4
V-VI-VII	3	5
V-VI-VII-VIII	4	3
V-VII	2	3
V-VII-VIII	3	7
V-VIII	2	12
VI	1	7
VI-VII	2	4
VI-VII-VIII	3	1
VI-VIII	2	1
VII	1	22
VII-VIII	2	17
VIII	1	24

Total species		879

See Table 1 for roman numerals, which refer to biomes. No biomes, number of inhabited biomes; Sp., species number

The most frequent combinations are I (147 species), I-II-II/III (118), I-II (112) and II (104). Other frequent combinations are I-II-II/III-V (54), I-II-V (26), V (24), VIII (24), II-II/III (23), VII (22), II/III (18), VII-VIII (17), III (16), V-VIII (12), II-V (12) and I-V (11). Therefore, the most frequent combinations among South American mammals imply few biomes, and these are the biomes that suffered most fragmentation during the Milankovitch cycles.

The four most eurybiomic species of the continent (BSI = 9) belong to three different orders and have the same climatic combination (I-II-II/III-III-IV-V-VI-VII-VIII), inhabiting all the biomes present in South America. We should stress that, although this combination is the same for all of them, each species inhabits in different areas and their distribution is latitudinal or altitudinal around the Andes. So, their comparison is limited. These species are the red bat (*Lasiurus borealis*), big brown bat (*Eptesicus fuscus*), puma (*Puma concolor*) and mountain vizcacha (*Lagidium viscacia*). Both chiropterans are vespertilionoid insectivores taking a wide variety of flying arthropods [51]. Thus, their high BSI may be related to their origin as North American immigrants. The puma is a very adaptable carnivorous species that can find food easily in any biome [52], tolerating almost any type of environment and landscape [53]. Finally, the mountain vizcachia is a rodent with a generalist diet that allows it to eat almost any kind of plant, including lichens, moss, and grass [32]. Biome generalist species stand out for ecological versatility, either latitudinal like puma which has an extension from Canada (64° N) to the South of Argentina (53° S) crossing all climatic zones, or altitudinal like the guanaco (*Lama guanicoe*), ranging from sea level to an elevation of 4000 m [32] and with a BSI = 7.

All South American biomes show very similar values for the ratio between the number of extreme eurybiomic species and the number of extreme eurybiomic climatic combinations (Table 7). One interesting issue arises when we compare our results about extreme generalist species (BSI ≥ 5) in South America with those for Africa [7]. To compare with Africa we study the 170 macromammalian species in South America, of which only thirteen species are extreme generalists (7.6 %). The evergreen tropical rainforest (I) in Africa had a lower number of extreme eurybiomic species per climatic combination (1.6) than the other biomes in the same continent (all around 2.5). However, in South America our results are very different; there is a very low and similar number of eurybiomic species per climatic combination in all biomes, approximately 1 (Table 7). These results appear to be against the previous interpretation of a possible lesser ecological overlapping among generalists in the rainforest than in other biomes [7]. This might be due to the fact that caviomorph rodents are included among micromammals but the body size of many of their species is comparable to the one in macromammals. Therefore, they might be occupying ecological niches that are occupied by some African ruminants. In this case, the late Quaternary extinction event might have influence on this pattern only if there was a selective extinction of extreme eurybiomic species (BSI ≥ 5), which seems improbable because most of the extinct South American large mammals were adapted to open environments instead of being very generalist species.

**Table 7:** Number of extreme eurybiomic species (BSI ≥ 5) in south American biomes

	All species			Macromammalia		
		
Biome	sp (BSI ≥ 5)	N.cc. (BSI ≥ 5)	sp/n.cc	sp (BSI ≥ 5)	N.cc. (BSI ≥ 5)	sp/n.cc
I	23	16	1.40	7	6	1.16
II	34	24	1.42	11	10	1.10
II/III	37	27	1.37	12	11	1.09
III	24	17	1.41	8	8	1.00
IV	23	15	1.53	9	8	1.12
V	34	25	1.36	12	11	1.09
VI	21	14	1.50	9	8	1.12
VII	30	22	1.36	12	11	1.09
VIII	23	17	1.35	8	8	1.00

sp., number of species with BSI ≥ 5; N.cc., number of climatic combinations with BSI ≥ 5; sp/n.cc., number of species per combination.

## Conclusion

The resource-use hypothesis [4,5] explains a great deal of the habitat theory [1,2], which downplays the role of biotic interactions, like predation or competition, as initiating causes of extinctions and speciation events. It suggests as the main promoters of speciation and extinction the physical environmental changes due to tectonics and global climatic change [1], which through the associated vicariance effect induce to the speciation. Therefore, habitat and evolutionary changes are joined [3]. Several recent works have obtained results in agreement with this theory. Jaramillo et al. [54] showed that plants diversity in the tropics is variable through time and correlates with long-term global climatic changes. Similar conclusions have been attained by van Dam et al. [55] when studying the changes in richness of the rodent faunas from the Iberian Neogene.

Although our results on the South American mammal assemblage are roughly concordant with the premises of the resource-use hypothesis, we have found striking differences with a previous study based on the African large mammal fauna [7]. To explain these differences between Africa and South America we should pay attention to three different aspects. First, the evolutionary and biogeographic histories of both continents are very different. South America's isolation during most of the Cenozoic and the late timing of connection with North America through the Panamanian land-bridge [56], which acted as an ecological filter that only favored the dispersal of generalist species or species specialized in savanna biome [1], provoked a situation of unbalance in the modern assemblage of South American mammals. This unbalance appears to be stronger in large herbivorous mammals than in other groups, because autochthonous ungulates disappeared in a large extent from South America due to changes in the continental environmental setting, and to the influence of the Pleistocene megafaunal extinction. This event was stronger in this continent with respect to Africa [46]. Second, the Andean orogeny and sub-Andean activity have helped to create a landscape of great elevational, climatic, and edaphic complexity, especially in Western Amazonia [57]. The Andes mountain range has many elevations along the continent with differential environments from its nearby lowlands. Thus, the elevation of the Andes is responsible for the high number of climatic combinations in South America in comparison with Africa, which has much less mountain ranges in its geography. Higher heterogeneity associated to mountain ranges is usually associated to higher species richness [58,59]. Third, small species show a behavior much more prone to specialization whatever the biome they inhabit, which is probably related to their higher reproductive rates, energetic and physiological constraints and generally smaller geographical distributions. Finally, although data on biomic specialization of species extinct during the late Quaternary megafaunal extinction event are not available, it seems improbable that its effect on the general pattern that we have shown here was significant enough to modify any of our conclusions, despite of the importance of this event on the assembly (or disassembly) of the modern South American mammalian fauna.

## Methods

### Data

The study area was the South American landmass. It excludes all offshore islands. The data represent the geographical distributions of all the modern terrestrial mammals occurring within South America; 709 micromammal species (Didelphimorphia, Paucituberculata, Microbiotheria, Insectivora, Chiroptera, Rodentia and Lagomorpha) and 170 macromammals (Xenarthra, Primates, Carnivora, Perissodactyla and Artiodactyla). The extinct species and species introduced by humans were omitted. For taxonomic consistency, we followed the species-level taxonomy of Wilson & Reeder [60].

Distribution ranges for the species were obtained mainly from the literature [33,51,61] and completed with Nowak [32], Macdonald [62] and the data bases InfoNatura [63] and World Wildlife Fund [64] for the species not present in the previous sources. For the species whose geographic range leaves South America we also used Hall [65].

### Measure of biomic specialization

There exist diverse measures of ecological specialization, as number of habitats occupied by a taxon, number of types of food the taxon uses, body mass, and number of subtaxa per taxon (see references in [7]). However, the resource-use hypothesis suggests some predictions which require a measure linked to the biomes where a species inhabits [1]. Thus, we followed Hernández Fernández and Vrba [7], which suggested the biomic specialization index (BSI) as a new specialization measure that can be used at the global scale or in different taxa.

### Climatic typology

We used the climatic classification of Walter [66], summarized in Table 1, which show 10 climatic zones that were mapped in Allué Andrade [67]. We use the terms biome and zonobiome synonymously, and we recognize that there is a one-to-one correspondence between these and the climatic zones (Table 1). South America has eight latitudinal climatic zones today, I-VIII.

**Figure 8:**
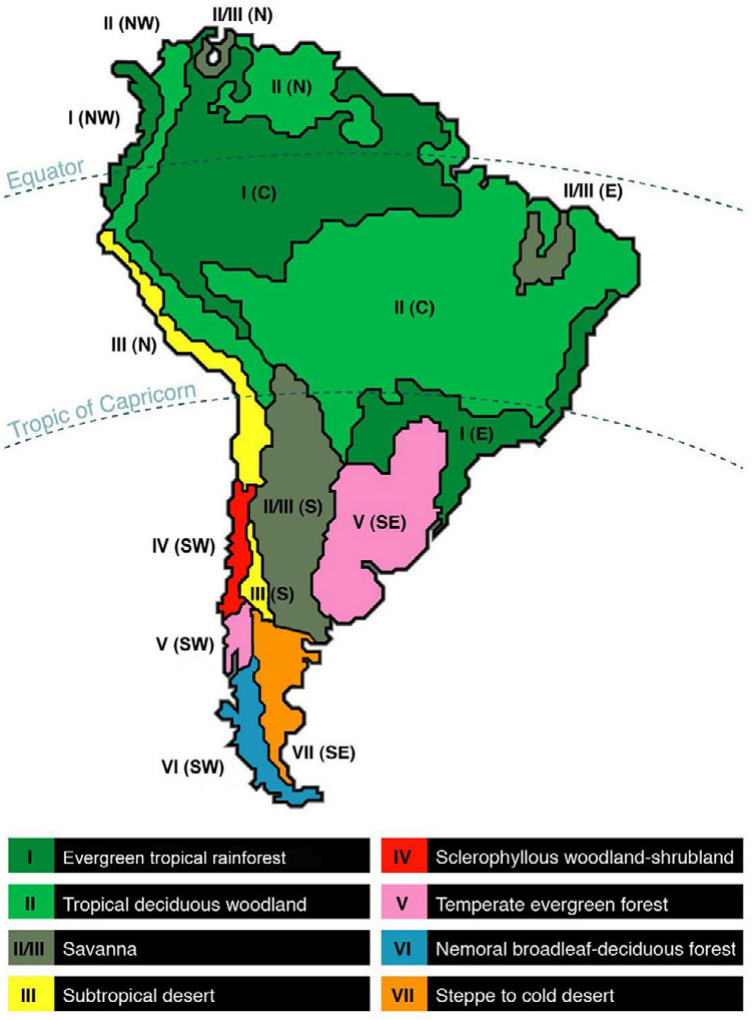
**Map of South American climatic dominions**. Modified after [67]. Abbreviations as in Table 8.

Sixteen climatic dominions, which are continuous terrestrial areas within one climate zone only [68], have been determined for the South American continent (Table 8, Figure 8). Since the altitudinal gradient represents a habitat series analogous to that of biomes in a latitudinal gradient [69-71], we also took into account latitudinal and altitudinal climatic zones.

**Table 8:** South American climatic dominions

Abbr.	Name	Climate zone
I (C)	Amazon rainforest	I
I (NO)	Colombian Choco rainforest	I
I (E)	Brazilian Atlantic forest	I
II (NO)	Andean Tropical dry forest	II
II (C)	Brazilian Cerrado	II
II (N)	Llanos of Venezuela	II
II/III (E)	Caatinga shrubland	II/III
II/III (S)	Chaco and Pampa grassland and Shrubland	II/III
II/III (N)	Maracaibo shrubland	II/III
III (N)	Atacama desert	III
III (S)	Monte desert	III
IV (SO)	Sclerophyllous Chilean shrubland	IV
V (SE)	Guarani subtropical forest	V
V (SO)	Valdivian warm temperate forest	V
VI (SO)	Magellanic subpolar forest	VI
VII (SE)	Patagonian grassland	VII

See Figure 8 for abbreviations.

### Bioclimatic characterization of species

The degree of biomic specialization of all South American mammals was investigated using the biomic specialization index (BSI) developed by Hernández Fernández and Vrba [7], which is the number of climate zones (biomes) inhabited by it. Thus, for most specialized species BSI equals 1, whereas for generalist species it could be as high as 10. The number of climate zones inhabited by a species is determined by the relative size of its geographical range [68]. If 15% or more of the geographical range of a species is situated within a climate zone, the species is considered to occupy that climate zone. It is also considered to occupy a specific climate zone when the species inhabits 50 % or more of one climatic dominion. The occurrence of each species in a climate zone or climatic dominion was determined by overlaying the mapped species distributions onto a base map of these biogeographical regions. Where a species occurred only very marginally in a region, it was not included. Finally, the presence in a mountain vegetation belt was also recorded as presence in the corresponding analogous climate zone [7].

We define biomic specialists, or stenobiomic species, as those with a BSI = 1. Thus generalist species are those with a BSI > 1. This latter category may be subdivided in two groups [7]: "semi-eurybiomic species" for those species with 1 < BSI < 5; and "extreme eurybiomic species" are those with BSI ≥ 5. BSI = 5 is considered the limit between semi-euribiomic and extreme eurybiomic species because those species that are able to inhabit five or more different biomes must confront very different environment conditions both in terms of temperature (e.g., from tropical rainforest to temperate evergreen forest) and rainfall (e.g., from rainforest to desert).

### Analyses

#### Monte Carlo

Using Monte Carlo simulations we tested the prediction that a non-random process has generated significantly more biomic specialist species than eurybiomic species. We set up a null hypothesis, which assumes that the observed presences-absences of each species are randomly placed among biomes. Nevertheless we fixed the number of species in each biome as the observed in South America today [7]. This process was repeated 1000 times for the total number of species in order to obtain null distributions of the frequency estimates for the percentage of species at each BSI.

To test whether extreme biomes have a higher proportion of biomic specialist species than the rest of biomes, the Monte Carlo simulations were employed too. The null hypothesis states that any difference between the proportion of stenobiomic species (BSI = 1) in each biome could have been resulted by chance.

#### Climatic combinations

We studied the different combinations of biomes that are today inhabited by the South American mammals and recorded the number of species in each of these combinations. The potential total number of climatic combinations (PTCC) that could be expected in the present world with 10 climate zones can be calculated with the formula:

$$\text{PTCC}=\sum_{i=1}^{10}\begin{array}{c} 10 \end{array}{\text{C}}_{\text{i}}=1+10+45+120+210+252+210+120+45+10=1023$$

In order to compare this potential number of climatic combinations with the observed frequencies in the South American mammals, we calculated a χ^2^.

## Authors' contributions

AMB and AAR conducted the gathering of data and performed all the data analyses. AMB developed the idea for the manuscript, wrote and co-edited all drafts, and prepared the final version of the manuscript. AAR co-edited the final drafts. MHF conceived, designed and coordinated the study, initiated the project, facilitated the gathering of contributors, co-refined the intellectual content, co-edited all drafts, and coordinated the authorship survey. AMB and MHF are the guarantors for the integrity of the article as a whole. EO-J and JM co-edited the final drafts and co-refined the intellectual content and scope. All authors read and approved the final manuscript.
